# Short-term mortality after perforated or bleeding peptic ulcer among elderly patients: a population-based cohort study

**DOI:** 10.1186/1471-2318-7-8

**Published:** 2007-04-17

**Authors:** Steffen Christensen, Anders Riis, Mette Nørgaard, Henrik T Sørensen, Reimar W Thomsen

**Affiliations:** 1 Department of Clinical Epidemiology, Aalborg and Aarhus Hospital, Aarhus University Hospital, Denmark; 2 Department of Epidemiology, School of Public Health, Boston University, MA, USA; 3 Department of Medicine V, Aarhus University Hospital, Denmark

## Abstract

**Background:**

Mortality after perforated and bleeding peptic ulcer increases with age. Limited data exist on how the higher burden of comorbidity among elderly patients affects this association. We aimed to examine the association of age with short-term mortality after perforated and bleeding peptic ulcer and to determine the impact of comorbidity on this association.

**Methods:**

In this population-based cohort study in three Danish counties between 1991 and 2003 we identified two cohorts of patients: those hospitalized with a first-time discharge diagnosis of perforated peptic ulcer and those with bleeding peptic ulcer. The diagnoses were ascertained from hospital discharge registries and mortality through the Danish Civil Registration System. Information on comorbidity and use of ulcer-related drugs was obtained through administrative medical databases. We computed age-, gender- and comorbidity-standardized 30-day mortality rates and used Cox's regression to estimate adjusted 30-day mortality rate ratios (MRR) for elderly compared with younger patients.

**Results:**

Among 2,061 patients with perforated peptic ulcer, 743 (36%) were 65–79 years old and 513 patients (25%) were aged 80+ years. Standardized 30-day mortality was 8.9% among patients younger than 65 years rising to 44.6% among patients aged 80+ years, corresponding to an adjusted MRR of 5.3 (95% CI: 4.0–7.0). Among 7,232 patients with bleeding peptic ulcer 2,372 (33%) were aged 80+ years. Standardized 30-day mortality among patients younger than 65 was 4.3% compared with 16.9% among patients aged 80+ years, corresponding to an adjusted MRR of 3.7 (95% CI: 2.9–4.7). Analyses stratified by comorbidity consistently showed high MRRs among elderly patients, regardless of comorbidity level.

**Conclusion:**

Ageing is a strong predictor for a poor outcome after perforated and bleeding peptic ulcer independently of comorbidity.

## Background

The incidence of complicated peptic ulcer disease (PUD), including perforated and bleeding peptic ulcer, increases with advanced age [1,2]. This increase has been attributed to the high frequency of risk factors for PUD among elderly patients, *e.g., Helicobacter pylori *colonization or use of non-steroidal anti-inflammatory drugs (NSAIDs) [3].

Perforated peptic ulcer is a serious condition with an overall reported mortality of 5%–25%, rising to as high as 50% with age [4-6]. Being closely related to advanced age, increased burden of comorbidity may partially explain the higher mortality among elderly patients. Nevertheless, virtually no data exist on the influence of comorbidity on age-related increase in mortality of perforated peptic ulcer.

Despite improvements in the last decades, in monitoring and treatment, bleeding peptic ulcer carries an unchanged short-term mortality approaching 10% [7,8]. Previous studies reported a higher short-term mortality from acute upper gastrointestinal bleeding including – but not confined to – bleeding peptic ulcer, among elderly compared with younger patients [9-11]. Again, no study has examined whether this association between age and mortality is related to increased burden of comorbidity in elderly patients.

We conducted this population-based cohort study to examine to which extent advanced age increased 30-day mortality of perforated and bleeding peptic ulcer and to determine the impact of comorbidity on this association.

## Methods

This population-based cohort study used data from medical registries in the Danish counties of North Jutland, Viborg and Aarhus, covering a population of 1.4 million, which is approximately 25% of the entire Danish population.

The Danish National Health Service provides tax-supported healthcare for all residents, guaranteeing free access to all general practitioners and hospitals and refunding a variable proportion of costs of prescribed medications. In Denmark all acute surgical conditions, including perforated and bleeding peptic ulcer, are treated at public hospitals operating under the authority of the National Health Service.

The different study periods were determined by availability of computerized prescription data records in the three counties, starting from January 1, 1991 in North Jutland, January 1, 1996 in Aarhus, and January 1, 1998 in Viborg County, through December 31, 2003 in all counties.

### Patients with perforated peptic ulcer and bleeding peptic ulcer

Using hospital discharge registries that are merged into a research database hosted by the Department of Clinical Epidemiology, Aarhus University Hospital, Denmark, we identified all patients with a first-time discharge diagnosis of perforated or bleeding peptic ulcer during the study period. The discharge registries, established in 1977, contain information on patients' civil registry numbers, dates of admission and discharge and up to 20 discharge diagnoses coded by physicians according to the International Classification of Diseases (ICD) (8^th ^edition until the end of 1993, 10^th ^edition thereafter). The discharge registries do not contain data on whether perforated or bleeding peptic ulcer was the reason for hospitalisation or whether it occurred as a complication during a hospitalisation with other diseases. ICD-8 codes utilized for identification of patients with perforated peptic ulcer were 53100, 53101, 53108, 53109, 53209, 53309, and 53409. The ICD-10 codes were K251, K252, K255, K256, K261, K262, K265, K266, K271, K272, K275, K276, K281, K282, K285, and K286. ICD-8 codes utilized for identification of patients with bleeding peptic ulcer were 53190, 53192, 53195, 53290, 53390, 53490 and ICD-10 codes were K250, K254, K260, K264, K270, K274, K280, and K284.

### Age and mortality

All Danish citizens are assigned a unique civil registry number encoding age, gender, and date of birth and death or migration. This number is included in all Danish administrative registries and enables unambiguous linkage between these [12].

To obtain information on death or migration, we linked the study cohort to the Danish Civil Registration System, which has kept records for the entire Danish population since 1968 on vital status [dead, alive], date of death, residence, and migration.

### Comorbidity

We used the Charlson comorbidity index as a summary measure for burden of comorbidity [13]. The Charlson index includes 19 major disease categories, including cardiovascular diseases, diabetes, chronic pulmonary, liver, renal and peptic ulcer disease, and solid and haematological malignancies. For computing the Charlson index score, a weight is assigned to each disease category, and the score is the sum of these weights. The index has been adapted and validated for use with hospital discharge registry data in ICD-based databases for the prediction of short- and long-term mortality [14]. For each patient we identified, in the hospital discharge registries, all primary and secondary discharge diagnoses for all hospitalisations preceding the date of the first hospitalisation for complicated peptic ulcer; from these data we computed the Charlson index score [15]. We defined 3 levels of comorbidity: low (index score of 0); moderate (index score of 1–2); and high (index score of more than 2). For this study, diagnoses of perforated and bleeding peptic ulcer were excluded from the Charlson index, since they defined our study population. Diagnoses of previous uncomplicated peptic ulcer disease (excluding peptic ulcer bleeding and perforation) were also removed from the index and included as a separate variable in the analyses.

### Other covariates

Information on use of drugs potentially associated with both advanced age and a poor outcome of bleeding and perforated peptic ulcer was obtained through the prescription databases of Viborg, North Jutland, and Aarhus counties. The prescription databases contain information on all prescriptions redeemed in any pharmacy in the county, including patient's civil registry number, the type and amount of drug prescribed and the prescription date [16]. By computerized data linkage we ascertained whether patients hospitalized with perforated peptic ulcer had redeemed prescriptions for oral glucocorticoids, low- or high-dose aspirin and/or NSAIDs within 60 days of admission. For patients hospitalized with bleeding peptic ulcer we retrieved information on redeemed prescriptions for oral glucocorticoids, aspirin, NSAIDs, vitamin K antagonists, calcium channel blockers, and antidepressants within 60 days of admission.

With the exception of aspirin and low-dose (200 mg) ibuprofen (which accounts for only 14% of ibuprofen use in Denmark), all included drugs are available in Denmark on the prescription-only basis [17].

### Statistical analysis

Based on the date of first admission with perforated or bleeding peptic ulcer we constructed Kaplan-Meier survival curves and life-table estimates of 30-day mortality for the main study variables: age group (15–64, 65–79, 80+ years), level of comorbidity (according to the Charlson score categories), gender, use of ulcer-related drugs, and previous hospitalisation with uncomplicated peptic ulcer disease. To compare 30-day mortality in the three different age groups we first computed standardized 30-day mortality rates, using direct standardization in the oldest age groups (65–79 years and 80+ years) to the distribution of comorbidity, and gender in the youngest age group (15–64 years). Second, we used Cox's regression analysis to estimate 30-day mortality rate ratios (MRRs), while adjusting for comorbidity level, gender, use of ulcer-related drugs, and previous hospitalisation with uncomplicated peptic ulcer disease. To examine the effect of comorbidity on the association between advanced age and 30-day mortality, we then stratified the analyses by the three levels of comorbidity. The Cox's regression analyses were repeated after including comorbidity diagnoses made during the index hospitalisation with complicated peptic ulcer. To examine whether age-related mortality changed during the study period we also stratified the analyses on calendar year band (1991–1997 vs. 1998–2004).

The assumption of proportional hazards in the Cox's regression model was assessed graphically and found appropriate.

All analyses were performed using SAS version 8.2 (SAS Institute Inc, Cary, NC).

The study was approved by the Aarhus University Hospital Registry Board and by the Danish Data Protection Agency.

## Results

### Perforated peptic ulcer

Descriptive data are provided in table 1. We identified 2,061 patients with a first-time discharge diagnosis of perforated peptic ulcer; of these 1,356 patients (61.4%) were more than 65 years old and 513 patients (25.7%) were aged 80+ years. A total of 38.5% of patients had at least one previous discharge diagnosis included in the Charlson comorbidity index and the burden of comorbidity increased with age. The majority of elderly patients (74.6% and 81.3% of the 65–79 and 80+ year old, respectively) were current users of NSAIDs, low- or high-dose aspirin or oral glucocorticoids.

**Table 1:** Characteristics of 2,061 patients with perforated and 7,232 patients with bleeding peptic ulcer in the counties of North Jutland, Aarhus and Viborg, Denmark.

	Perforated peptic ulcer			Bleeding peptic ulcer		
	**15–64 years** N	**65–79 years** N	**80+ years** N	**15–64 years** N	**65–79 years** N	**80+ years** N

**Gender**						
Female	338 (42.0%)	410 (55.2%)	358 (69.8%)	739 (35.5%)	1329 (47.1%)	1402 (59.1%)
Male	467 (58.0%)	333 (44.8%)	155 (30.2%)	1347 (64.6%)	1445 (52.1%)	970 (40.9%)
Ulcer-related drugs*						
Use†	484 (60.1%)	554 (74.6%)	417 (81.3%)	1434 (68.7%)	2093 (75.4%)	1950 (82.2%)
No use	321 (39.9%)	189 (25.4%)	96 (18.7%)	652 (31.3%)	681 (24.6%)	422 (17.8%)
**Previous uncomplicated peptic ulcer disease**						
No	709 (88.1%)	657 (88.4%)	463 (90.3%)	1829 (87.5%)	2446 (88.2%)	2115 (89.2%)
Yes	96 (11.9%)	86 (11.6%)	50 (9.7%)	257 (12.6%)	328 (11.8%)	257 (10.8%)
**Comorbidity**#						
Low	616 (76.5%)	378 (50.9%)	273 (53.2%)	1442 (68.2%)	1322 (47.7%)	1148 (48.4%)
Moderate	161 (20.0%)	290 (39.0%)	208 (40.6%)	523 (25.1%)	1139 (41.0%)	1013 (42.7%)
High	28 (3.5%)	75 (10.1%)	32 (6.2%)	141 (6.7%)	313 (11.3%)	212 (8.9%)

*For perforated peptic ulcer "ulcer-related drugs" are: oral glucocorticoids, NSAIDs and aspirin. For bleeding peptic ulcer "ulcer-related drugs" are: non-aspirin NSAIDs, aspirin, oral glucocorticoids, vitamin K antagonists, calcium channel blockers, and/or antidepressants

† Use = filled prescription 60 days before admission with perforated peptic ulcer.

# Three levels of the index were defined: Low (no co-morbidity), medium (Charlson index 1–2) and high (Charlson index 2+).

**Figure 1:**
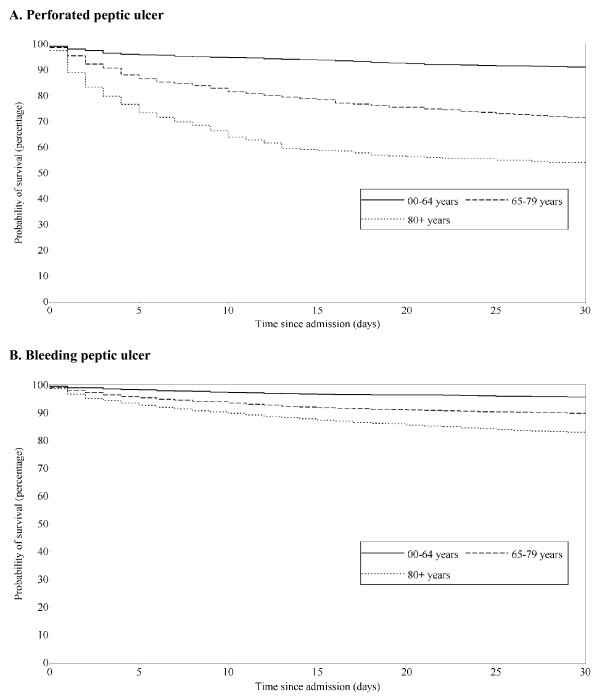
Survival of patients with perforated (A) and bleeding peptic ulcer (B) according to age group. Aarhus, North Jutland and Viborg counties, Denmark.

Overall 30-day mortality from perforated peptic ulcer was 25.3%, but it increased from 8.9% among patients younger than 65 years to respectively 28.5% and 46.0% among patients aged 65–79 years and 80+ years (table 2). The standardized 30-day mortality was 24.2% among patients aged 65–79 years and 44.6% among those aged 80+ years. As seen in figure 1, the higher mortality among elderly patients was most pronounced during the first 2 weeks of hospitalisation with perforated peptic ulcer. The adjusted 30-day MRRs were 2.8 (95% CI: 2.1–3.6) among patients aged 65–79 years and 5.3 (95% CI: 4.0–6.9) among patients aged 80+ years, each compared with patients younger than 65 years (table 2). Including comorbidity diagnoses made during the index hospitalisation with perforated peptic ulcer did not change any of the MRRs. Further, we found no difference in age-related MRR between calendar year bands, 1991–1997 and 1998–2004 (data not shown). Regardless of comorbidity level, MRRs were consistently higher among elderly than among younger patients (table 3). As seen from table 3 the MRRs associated with increased age were highest among patients with low comorbidity levels. By contrast, the absolute differences in 30-day mortality between the three age groups were similar across the comorbidity index groups.

**Table 2:** Crude and standardized 30-day mortality for patients with a first-time discharge diagnosis of perforated or bleeding peptic ulcer and 30-day mortality rate ratios (MRRs) relative to patients aged 15–64.

Age groups	Number of patients N	30-day mortality	Standardized 30-day mortality ∞ (95% CI)	Crude 30-day MRR (95% CI)	Adjusted 30-day MRR# (95% CI)
**Perforated peptic ulcer**					
15–64	805	8.9%	8.9% (7.0%–10.9%)	1 (ref)	1 (ref)
65–79	743	28.5%	24.2% (20.7%–27.6%)	3.5 (2.7–4.6)	2.8 (2.1–3.6)
80+	513	46.0%	44.6% (38.8%–50.5%)	6.6 (5.1–8.6)	5.3 (4.1–7.0)
**Bleeding peptic ulcer**					
15–64	2086	4.3%	4.3% (3.4%–5.2%)	1 (ref)	1 (ref)
65–79	2774	10.2%	8.7% (7.6%–9.9%)	2.4 (1.9–3.1)	2.2 (1.7–2.8)
80+	2372	17.0%	16.9% (15.0%–18.7%)	4.2 (3.3–5.3)	3.7 (2.9–4.7)

∞ Standardized to the distribution of gender and comorbidity in the youngest age group.

# Adjusted by Cox regression analysis for gender, comorbidity prior to hospitalisation with complicated peptic ulcer, use of ulcer-related drugs and previous admissions with uncomplicated peptic ulcer.

**Table 3:** 30-day mortality and mortality rate ratios (MRR) for patients with perforated and bleeding peptic ulcer stratified by level of comorbidity.

**Comorbidity score **Ω	Age (years)	Number of patients	30-day mortality	Crude 30-day MRR (95% CI)	Adjusted 30-day MRR* (95% CI)
***Perforated Peptic ulcer***					

Low	15–64	616	6.0%	1 (ref)	1 (ref)
	65–79	378	20.9%	3.8 (2.5–5.6)	3.5 (2.3–5.1)
	80+	273	41.0%	8.6 (5.9–12.4)	8.1 (5.5–11.9)
Moderate	15–64	161	16.8%	1 (ref)	1 (ref)
	65–79	290	34.8%	2.3 (1.5–3.4)	2.1 (1.4–3.2)
	80+	208	50.0%	3.8 (2.5–5.7)	3.4 (2.3–5.3)
High	15–64	28	28.6%	1 (ref)	1 (ref)
	65–79	75	42.7%	1.6 (0.8–3.6)	1.6 (0.7–3.6)
	80+	32	62.5%	3.0 (1.3–6.7)	2.9 (1.2–6.8)

***Bleeding Peptic ulcer***					

Low	15–64	1422	3.4%	1 (ref)	1 (ref)
	65–79	1322	6.7%	2.0 (1.4–2.9)	1.9 (1.3–2.7)
	80+	1148	16.0%	4.9 (3.6–6.8)	4.5 (3.3–6.2)
Moderate	15–64	523	5.5%	1 (ref)	1 (ref)
	65–79	1139	13.4%	2.5 (1.7–3.7)	2.5 (1.7–3.7)
	80+	1012	16.9%	3.2 (2.2–4.8)	3.2 (2.2–4.8)
High	15–64	141	9.2%	1 (ref)	1 (ref)
	65–79	313	13.7%	1.5 (0.8–2.8)	1.5 (0.8–2.7)
	80+	212	25.0%	2.9 (1.6–5.1)	2.7 (1.5–4.9)

Ω Three levels of the index were defined: Low (no co-morbidity), medium (Charlson Index 1–2) and high (Charlson Index 3+). * Adjusted in a Cox regression model for gender, previous admissions with uncomplicated peptic ulcer and ulcer-related drug use.

### Bleeding peptic ulcer

We identified 7,232 patients with a first-time discharge diagnosis of bleeding peptic ulcer, of whom 5,146 (71.2%) were older than 65 years of age and 2,372 (32.7%) were aged 80+ years. The prevalence of patients with comorbidities increased from 31.5% among patients younger than 65 years to 51.6% among patients aged 80+ years (table 1).

Overall 30-day mortality of bleeding peptic ulcer was 10.8%, but rose from 4.3% among patients younger than 65 years to 10.2% and 17.0% among patients aged 65–79 years and 80+ years, respectively (table 2, figure 1). After standardization, the 30-day mortality decreased to 8.7% among the 65–79 year old and to 16.9% among those aged 80+ years. Adjusted MRRs were 2.2 (95% CI: 1.7–2.8) and 3.7 (95% CI: 2.9–4.7) among patients aged 65–79 years and 80+ years compared with patients younger than 65 years. Including comorbidity diagnosis made during the index hospitalisation with bleeding peptic ulcer did not change any of the MRRs and we found no difference in age-related MRRs between calendar year bands, 1991–1997 and 1998–2004 (data not shown). The stratified analyses consistently showed higher MRRs among elderly patients compared with younger patients regardless of level of comorbidity (table 3). Again the MRRs associated with increased age were highest among patients with low comorbidity level whereas the absolute differences in 30-day mortality among the three age groups were similar across the three strata of comorbidity.

## Discussion

In this population-based cohort study of more than 9,000 patients we found that elderly patients had substantially increased 30-day mortality of perforated and bleeding peptic ulcer compared with younger patients. The mortality increases were most pronounced during the first 14 days following hospitalisation, particularly among patients with perforated peptic ulcer. An increased burden of comorbidity among elderly patients did not explain the association between advanced age and increased mortality, with the strongest association observed among patients with no history of hospital-diagnosed comorbidity.

The findings of a higher mortality of perforated and bleeding peptic ulcer among elderly patients corroborate results of most previous studies [18-21]. However, use of ulcer-related drugs and comorbidity may have influenced results of these studies making them difficult to interpret. No previous studies have focused on age-related increasing level of comorbidity influencing the effect of advanced age on the outcome after complicated peptic ulcer. Two recent population-based studies from Scotland and the Netherlands found that after adjustment for multiple confounders, age remained an independent predictor of death from upper gastrointestinal bleeding [7,10]. In contrast, Segal et al. [22] and Antler et al. [23] found similar mortality rates of upper gastrointestinal bleeding among young and elderly patients in two observational US studies, in 1981 and 1996. It is important to recognize that these data are derived from studies of the outcome of upper gastrointestinal bleeding, which includes – but is not confined to – bleeding peptic ulcer. Upper gastrointestinal bleeding among young patients is more likely to be caused by lesions induced by excess alcohol consumption, *e.g*. oesophageal varices, Mallory-Weiss lesions, or hemorrhagic gastritis, whereas elderly patients are more likely to bleed from peptic ulcers [22,24]. Thus, the association between age and the outcome of upper gastrointestinal bleeding may be biased by the different spectrum of bleeding lesions. Few previous studies found comorbidity to be an independent prognostic factor for complicated peptic ulcer. A recent Dutch study based on retrospectively reviewed medical records found that 10 of 13 death following peptic ulcer bleeding were unavoidable primarily because of severe comorbidities [25].

The main strengths of the present study include its large size and the uniformly organized health system allowing a population-based design and the use of independent medical databases, which limits the risk of selection and information bias. A further advantage is the ability to adjust the analysis for the pre-hospitalisation use of ulcer-related drugs, which is an important potential confounding factor. However, residual confounding could stem from potential misclassification of drug use due to lack of compliance.

We adjusted for level of comorbidity by using the Charlson Comorbidity Index. Registration of comorbidities and therefore control of confounding might have been more accurate among younger patients; thus, residual confounding could cause overestimation of the relative mortality in elderly patients. Coding errors in the routine hospital discharge data might have lead to over-ascertainment of perforated or bleeding peptic ulcer. However, the positive predictive value of recorded gastrointestinal site-specific discharge diagnosis has been reported to be high [26], and as any misclassification is unlikely to be related to age the influence on our results should be negligible. Furthermore, the validity of the methodology used in this study is backed by a recent Danish government-supported national health care quality measurement project on outcome of bleeding and perforated peptic ulcer. Using prospectively collected clinical data, the project found an overall 30-day mortality very similar to ours, *i.e*. 28% vs. 25% for perforated and 11% vs.11% for bleeding peptic ulcer [27].

Comorbidity apparently cannot explain most of the mortality increase among elderly patients with complicated peptic ulcer disease. Other factors, such as diagnostic difficulties, treatment differences, clinical quality, as well as the progressive processes of functional and biological deterioration associated with advanced age may play a role [28]. Delayed surgical treatment is a major prognostic factor for poor outcome of perforated peptic ulcer [29] and treatment delay may be directly related to age [3,30]. Malnutrition is associated with decreased wound healing, increased length of hospital stay and increased mortality in surgical patients [31]. As the prevalence of malnutrition is markedly higher among elderly patients, it may help explain the increased mortality among elderly patients. Sepsis and multi-organ failure are reportedly leading causes of death in patients with perforated peptic ulcer [32]. Ageing is associated with complex changes in the immune system, including an altered acute phase response, and thus an increased risk of and poorer outcome after severe infections [33]. Unfortunately, the databases used in this study lack clinical data on treatment delay or the patients' nutritional status. We were unable to differentiate which complicated peptic ulcers were reasons for hospitalisation and which cases occurred during hospitalisation with other diseases. Further studies may enlighten the role of these factors in the mortality from complicated peptic ulcer disease in elderly patients.

## Conclusion

In conclusion, ageing is a strong predictor for a poor outcome of perforated and bleeding peptic ulcer independently of comorbidity.

## Competing interests

The author(s) declare that they have no competing interests.

## Authors' contributions

All authors participated in the design of the study, AR performed the statistical analyses, and all authors participated in the interpretation of the results. All authors helped drafting the manuscript and all read and approved the final manuscript.

## Pre-publication history

The pre-publication history for this paper can be accessed here:

http://www.biomedcentral.com/1471-2318/7/8/prepub
